# Accuracy of Loop-Mediated Isothermal Amplification for Diagnosis of Human Leptospirosis in Thailand

**DOI:** 10.4269/ajtmh.2011.10-0473

**Published:** 2011-04-05

**Authors:** Piengchan Sonthayanon, Wirongrong Chierakul, Vanaporn Wuthiekanun, Janjira Thaipadungpanit, Thareerat Kalambaheti, Siriphan Boonsilp, Premjit Amornchai, Lee D. Smythe, Direk Limmathurotsakul, Nicholas P. Day, Sharon J. Peacock

**Affiliations:** Department of Clinical Tropical Medicine, Department of Molecular Tropical Medicine and Genetics, Mahidol-Oxford Tropical Medicine Research Unit, Department of Microbiology and Immunology and Department of Tropical Hygiene, Faculty of Tropical Medicine, Mahidol University, Bangkok, Thailand; World Health Organization/Food and Agriculture Organization of the United Nations/World Organisation for Animal Health Collaborating Centre for Reference and Research on Leptospirosis, Western Pacific Region, Communicable Disease Unit, Queensland Health Forensic and Scientific Services, Brisbane, Queensland, Australia; Center for Clinical Vaccinology and Tropical Medicine, Nuffield Department of Clinical Medicine, University of Oxford, Oxford, United Kingdom; Department of Medicine, University of Cambridge, Addenbrooke's Hospital, Cambridge, United Kingdom

## Abstract

There is a lack of diagnostic tests for leptospirosis in technology-restricted settings. We developed loop-mediated isothermal amplification (LAMP) specific for the 16S ribosomal RNA gene (*rrs*) of pathogenic and intermediate group *Leptospira* species. The lower limit of detection was 10 genomic equivalents/reaction, and analytical specificity was high; we observed positive reactions for pathogenic/intermediate groups and negative reactions for non-pathogenic *Leptospira* species and other bacterial species. We evaluated this assay in Thailand by using a case–control study of 133 patients with laboratory-proven leptospirosis and 133 patients with other febrile illnesses. Using admission blood, we found that the *rrs* LAMP showed positive results in 58 of 133 cases (diagnostic sensitivity = 43.6, 95% confidence interval [CI] = 35.0–52.5) and in 22 of 133 controls (diagnostic specificity = 83.5, 95% CI = 76.0–89.3). Sensitivity was high for 39 patients who were culture positive for *Leptospira* spp. (84.6, 95% CI = 69.5–94.1). The *rrs* LAMP can provide an admission diagnosis in approximately half of patients with leptospirosis, but its clinical utility is reduced by a lower specificity.

## Introduction

Leptospirosis is an acute febrile illness caused by pathogenic species belonging to the genus *Leptospira*. This zoonotic disease has a worldwide distribution, but has the greatest impact on health in developing countries where it is often grossly under-recognized. Clinical features are similar to a range of other infectious diseases that occur in the same geographic regions, including scrub typhus, dengue, and malaria.^1^ Accurate laboratory diagnosis of leptospirosis is important for correct patient management, but there is a paucity of diagnostic tests that provide timely information around the time of admission. Culture can be performed during the first week of symptoms but is rarely undertaken because it requires considerable expertise, is time-consuming, and may not provide a positive result for weeks or months.

The microscopic agglutination test (MAT) is commonly used as a serologic diagnostic gold standard. This test relies on detecting an increase in antibody titer between serum samples obtained at least two weeks apart and provides a retrospective diagnosis that is useful for epidemiologic purposes but does not benefit individual patient management. Conventional and real-time polymerase chain reactions have been described for detection of pathogenic *Leptospira* in blood at the time of patient presentation,^2^–^5^ and are useful for investigation of patients presenting within the first week of illness (during the leptospiremic phase of infection) in settings that have the necessary equipment and trained laboratory personnel. However, these assays are not in routine use in many countries where leptospirosis is highly endemic because the equipment required is costly, and performing the assay to the required standard demands considerable expertise and training.

Loop-mediated isothermal amplification (LAMP) is an alternative method of rapid DNA amplification. The final products are stem-loop DNAs with several inverted repeats of the target and cauliflower-like structures with multiple loops formed by annealing between alternately inverted repeats of the target in the same strand.^6^ The reaction results in the accumulation of 10^9^ copies of target and simply requires a laboratory water bath or heating block to maintain a constant temperature of 60–65°C, making it particularly suited to lower-technology settings. The LAMP has been developed for detection of a range of pathogenic bacteria and viruses,^7^–^9^ including *Leptospira* species.^10^ The published *Leptospira* assay targets *lipL41*, a gene encoding the outer membrane protein LipL41, which is expressed by pathogenic *Leptospira*.^11^ The lower limit of detection was reported to be approximately 100 genomic equivalents (GEs) per reaction, and the assay was specific for *Leptospira* in a laboratory evaluation. The assay detected *Leptospira* in seven mouse kidney samples obtained from animals captured during environmental surveillance,^10^ but has not undergone clinical evaluation to date.

*Leptospira* genomes contain two 16S ribosomal RNA genes for which there are multiple sequences of variable length in public databases, phylogenetic analysis of which has identified three clades within the genus composed of the pathogenic species, non-pathogenic species, and a clade with intermediate 16S ribosomal RNA gene sequence relatedness.^12^ The latter group is considered to represent opportunistic/intermediate pathogens, although there are an increasing number of reports associating them with human leptospirosis.^13^–^15^ The objective of this study was to use the phylogenetic distinctions inherent in the 16S ribosomal RNA gene sequence to develop an alternative LAMP assay (*rrs* LAMP) that can detect *Leptospira* spp. in the pathogenic and intermediate groups, and to undertake a clinical case–control evaluation of the diagnostic accuracy of *rrs* and *lipL41* LAMP in rural Thailand.

## Materials and Methods

### Laboratory strains.

The *rrs* LAMP was initially developed by using genomic DNA extracted from *L. interrogans* serovar Autumnalis strain L0551 isolated in 2001 from a patient in northeastern Thailand, and then further evaluated by using an additional 23 *Leptospira* isolates shown in Table 1. The specificity of the assay for *Leptospira* spp. was evaluated by using one clinical isolate of each of the following bacterial species: *Staphylococcus aureus, Enterococcus* sp*., Escherichia coli, Salmonella enterica* serovar Typhi, *Klebsiella pneumoniae, Pseudomonas aeruginosa, Burkholderia pseudomallei, Orientia tsutsugamushi* strain Kato, and *Rickettsia typhi*. Genomic DNA was extracted from laboratory cultures by using the Wizard^®^ Genomic DNA Extraction Kit (Promega, Madison, WI) with the addition of 5 μL of lysostaphin (10 mg/mL) during the extraction of *S. aureus* DNA.

### Primer design.

A total of 39 sequences for the full-length 16S ribosomal RNA gene of *Leptospira* spp. were downloaded from GenBank, of which 23 sequences were from 8 pathogenic *Leptospira* spp., 6 sequences were from 3 intermediate group *Leptospira* spp., and 10 sequences were from 6 non-pathogenic *Leptospira* spp. Accession numbers are pathogenic species *Leptospira interrogans* (n = 10, accession nos. AY631894, AY996790, AY996791, AY996792, AY996893, AY996794, AY996796, AY996797, AY9967698, AY99680), *L. borgpetersenii* (n = 2, accession nos. AY631884, AY887899), *L. kirschneri* (n = 3, accession nos. AY631895, AY996801, AY996802), *L. alexanderi* (n = 3, accession nos. AY631880, AY996803, AY996804), *L. santarosai* (n = 2, accession nos. AY631883, AY996805), *L. noguchii* (n = 1, accession no. AY631886), *L. alstonii* (n = 1, accession no. AY631881), *L. weilii* (n = 1, accession no. AY631877); intermediate species *L. inadai* (n = 3, accession nos. AY631887, AY631891, AY631896), *L. broomii* (n = 1, accession no. AY796065), *L. fainei* (n = 2, accession nos. AY631885, AY996789); and non-pathogenic species *L. biflexa* (n = 2, accession nos. AY631876, AY631893), *L. meyeri* (n = 3, accession nos. AY631878, AY631889, AY631892), *L. wolbachii* (n = 2, accession nos. AY631879, AY631890), *L. vanthielii* (n = 1, accession no. AY631897), *L. terpstrae* (n = 1, accession no. AY631888), and *L. yanagawae* (n = 1, accession no. AY631882). The partial 16S ribosomal RNA sequence of three recently described *Leptospira* spp. were also included: pathogenic *L. kmetyi* (n = 1, accession no. AB279549) and intermediate group *L. licerasiae* (n = 9, accession nos. EF612280-8) and *L. wolffii* (n = 1, accession no. EF025496).

**Figure 1.**
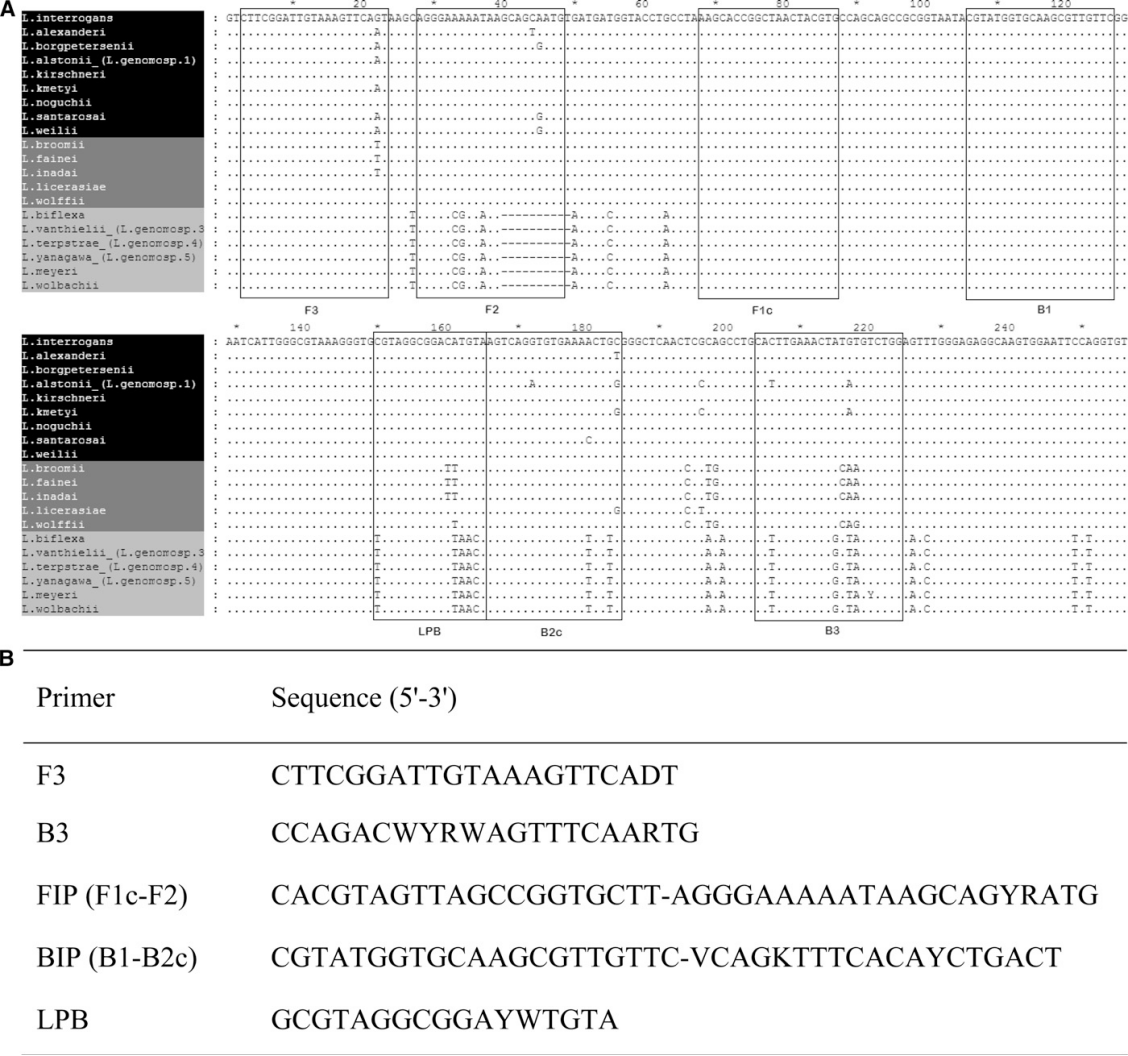
**A**, Sequence alignment of a region of the 16S ribosomal RNA gene (*rrs*) of 20 *Leptospira* spp. to demonstrate the regions selected for primer design. The objective was to design primers that annealed to the target gene of *Leptospira* spp. that belonged to either the pathogenic (**white letters in black box**) or intermediate group (**white letters in gray box**), but that failed to anneal to the target gene of *Leptospira* spp. in the non-pathogenic group (**black letters in gray box**). F1c, F2, F3, B1, B2c, B3, and LPB refer to the names and locations of target sequences for the primers used. **B**, Sequences of *rrs* loop-mediated isothermal amplification (LAMP) primers. Primer FIP consisted of the F1complementary sequence (20 nucleotides) and the F2 direct sequence (21 nucleotides). Primer BIP consisted of the B1 direct sequence (21 nucleotides) and the B2 complementary sequence (19 nucleotides).

All sequences were aligned and compared by using Clustal X version 1.83^16^ and Genedoc (http://www.nrbsc.org/gfx/genedoc/index.html).^17^ A set of five primers consisting of two outer primers (F3 and B3), two inner primers (forward inner primer FIP and backward inner primer BIP), and one loop primer (loop B (LBP)) were designed by using PrimerExplorer version 4 (http://primerexplorer.jp/e/). The target sequences were regions that were specific to the 16S ribosomal RNA gene sequence of all pathogenic and intermediate but not non-pathogenic *Leptospira* spp., as shown in Figure 1A. Primers are shown in Figure 1B.

### LAMP assays.

The *lipL41* LAMP was performed by using primers and conditions described.^10^ The *rrs* LAMP was carried out in a reaction mixture of 25 μL containing 5 pmol of each outer primer (F3, B3), 40 pmol of each inner primer (FIP, BIP), 20 pmol of loop primer (LPB), 1× ThermoPol reaction buffer, 0.8 M Betaine (Sigma, St. Louis, MO), 1.4 mM dNTP, 6 mM MgSO_4_, 8 units of *Bst* DNA polymerase, large fragment (New England Biolabs, Ipswich, MA), and 1 μL (approximately 20–100 ng) of genomic DNA extracted from laboratory cultures of *Leptospira* spp. The reaction mixture was incubated at 63°C for 2 hours and then heated at 80°C for 5 minutes to terminate the reaction. The presence of product was defined for both LAMP assays on the basis of detecting a white precipitate with the naked eye after centrifugation at 13,000 × *g* for 2 minutes. All results were confirmed by examining 3 μL of amplified products by using 2% agarose gel electrophoresis and staining with ethidium bromide. Positive and negative controls were genomic DNA from *L. interrogans* serovar Lai and reaction mixture without DNA, respectively. The analytical sensitivity of *rrs* LAMP was determined by using a 10-fold serial dilution (ranging from 2 × 10^6^ to 0.02 GEs) of *L. interrogans* serovar Lai genomic DNA. The analytical specificity of this assay was determined by using DNA from non-pathogenic *Leptospira* spp. and a range of other bacterial species.

### Clinical validation.

Patients with (cases) or without leptospirosis (controls) were selected from a prospective cohort of 418 patients with an acute febrile illness who came to the Udon Thani Regional Hospital in northeastern Thailand during January 10, 2001–June 16, 2002.^18^ Briefly, patients were recruited into the study during twice a day ward rounds. Inclusion criteria were patients who were ≥ 15 years of age with fever (> 37.8°C) of unknown cause who agreed to participate and to attend out-patient follow-up for a convalescent-phase serum sample. The exclusion criterion was the presence of a definable infection at the time of admission.

Blood was obtained on admission from all patients for *Leptospira* culture, serologic testing, and molecular diagnostics, and a second (convalescent-phase) sample was obtained for serologic testing approximately two weeks later. *Leptospira* culture was performed as described,^19^ and isolates sent to the World Health Organization/Food and Agriculture Organization of the United Nations/World Organisation for Animal Health Collaborating Centre for Reference and Research on Leptospirosis Collaborating Center for Reference and Research on Leptospirosis (Brisbane, Queensland, Australia) for serovar identification by using the cross-agglutinin absorption test.^20^ The MAT was performed by the World Health Organization/Food and Agriculture Organization of the United Nations/World Organisation for Animal Health Collaborating Centre for Reference and Research on Leptospirosis as described^20^ by using a live panel of antigens representing ubiquitous and locally prevalent serovars. A diagnosis of leptospirosis was based on isolation of *Leptospira* from blood and/or a positive MAT result, which was defined as a four-fold increase in titer between acute-phase and convalescence-phase samples or a single titer of ≥ 1:400. Patients who did not meet these criteria were defined as not having current or recent leptospirosis.

All patients diagnosed as having leptospirosis during the prospective cohort study (n = 133) were selected as cases. Laboratory confirmation was made on the basis of culture-positive and MAT-positive results in 21 (16%) patients, culture- positive results and MAT-negative results in 18 (13%) patients, and culture-negative results and MAT-positive results in 94 (71%) patients. A positive MAT result was based on a four-fold increasing titer in 97 cases and a single titer of ≥ 1:400 in 27 cases. Controls (n = 133) were randomly selected from those patients who did not meet the diagnostic criteria for leptospirosis. Patients in this group had a convalescent-phase serum sample obtained a median of 17 days (range = 10–43 days, interquartile range [IQR] = 13–21 days) after the onset of symptoms.

A database was created in which cases and controls were entered, randomized, and blinded to the technician prior to performing the two LAMP assays. DNA was extracted from a 5-mL blood sample (plus EDTA) obtained from cases and controls on admission and stored at –80°C prior to use. Extraction was performed by using the Nucleon BACC 3 Kit B (GE Healthcare Biosciences, Piscataway, NJ), and the extract was suspended in 1 mL of Tris-EDTA buffer. The LAMP assays were described as above except that 5 μL of DNA extracted from whole blood was used per reaction. All patient samples were evaluated in duplicate for each of the two LAMP assays. A positive result for either one or both samples was interpreted as positive.

The study protocol was reviewed and approved by the Ethics Committee of the Faculty of Tropical Medicine, Mahidol University, Bangkok, Thailand.

### Statistical analysis.

Statistical analyses were performed by using STATA/SE version 10.0 (StataCorp., College Station, TX). Diagnostic sensitivity (DSe) and specificity (DSp) of each PCR assay was defined against the combined result for culture and MAT (a positive result for either or both being interpreted as diagnostic for leptospirosis), and expressed as a proportion with exact 95% confidence intervals (CIs). Comparison of DSe and DSp for the two LAMP assays was performed by using the McNemar test. The DSe of each assay was re-evaluated in the subset of patients who were culture positive.

## Results

### Analytical sensitivity and specificity.

**Figure 2.**
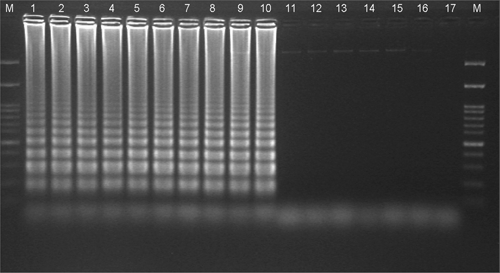
16S ribosomal RNA gene loop-mediated isothermal amplification products separated by 2% agarose gel electrophoresis. Lanes 1–8, pathogenic *Leptospira* spp. (*L. interrogans*, *L. kirshneri*, *L. noguchii*, *L. alexanderi*, *L. weilli*, *L. borgpetersenii*, *L. santarosai*, and *L. alstonii,* respectively); lanes 9 and 10, intermediate group *Leptospira* spp. (*L. inadai* and *L. wolffii*, respectively). No products were observed for non-pathogenic species (lanes 11–16, *L. biflexa*, *L. meyeri*, *L. wolbachii*, *L. terpstrae*, *L. yanagawae*, and *Turneriella parva*, respectively). Lane M, 100-basepair DNA marker; lane 17, negative (no template) control.

Primers were designed that were predicted to anneal to a region of *rrs* of *Leptospira* spp. belonging to pathogenic and intermediate groups but not to the non-pathogenic group. The analytical specificity of these primers was assessed using genomic DNA extracted from 15 *Leptospira* spp., including representatives of each of the three groups (Figure 2) demonstrates confirmation of the expected annealing pattern. Specificity was further evaluated by testing a range of bacterial species that commonly cause febrile illness in our patient population. No product was observed using genomic DNA from one isolate of each of the following bacteria: *S. aureus, Enterococcus* spp*., E. coli, S. enterica* serovar Typhi, *K. pneumoniae, P. aeruginosa, B. pseudomallei, O. tsutsugamushi* strain Kato, and *R. typhi*. The analytical sensitivity (lower limit of detection) determined by using genomic DNA extracted from pure laboratory culture of *L. interrogans* serovar Lai was equivalent to10 GEs/reaction.

### Diagnostic sensitivity and specificity of *rrs* LAMP.

**Figure 3.**
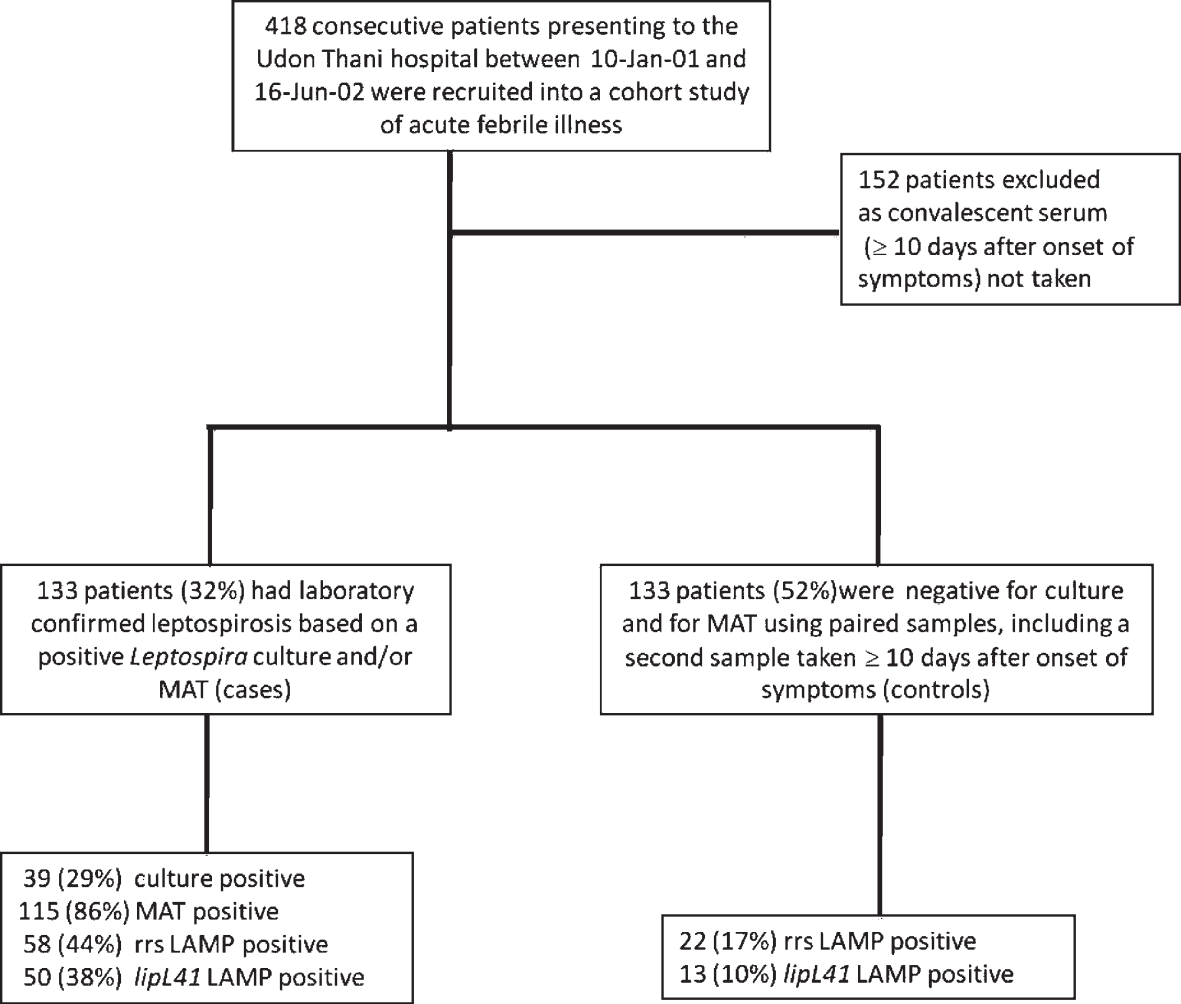
Patient recruitment and result of laboratory tests for leptospirosis.

In total, 133 patients with laboratory confirmed leptospirosis (cases) and 133 patients who did not have leptospirosis (controls) were evaluated. Patient recruitment and diagnostic testing are summarized in Figure 3. Of 133 patients, 18 (13%) were culture positive and MAT negative, 94 patients (71%) were MAT positive and culture negative, and 21 (16%) were MAT positive and culture positive. The median (IQR, range) age was 35 years (26–46, 15–74 years) for cases and 42 years (29–54, 15–79 years) for controls (*P* = 0.01). The median duration of illness prior to admission was 4 days (IQR = 2–5 days, range = 1–12 days) for cases and 6 days (IQR = 3–9 days, range = 0–33 days) for controls (*P* < 0.001). Most (120 of 133, 90.2%) of the case-patients came to the hospital with ≤ 7 days of the onset of symptoms. Fourteen case-patients (11%) had received antimicrobial drug therapy that would be predicted to be bacteriocidal for *Leptospira* prior to blood sample collection. The discharge diagnoses of controls were as follows: scrub typhus (n = 54), other bacterial septicemia (n = 8), *E. coli* (n = 2), *K. pneumoniae* (n = 2), *Acinetobacter baumanii* (n = 1), *Corynebacterium jeikeium* (n = 1), *Enterococcus* sp. (n = 1), *Streptococcus pneumoniae* (n = 1), dengue fever (n = 5), murine typhus (n = 4), melioidosis (n = 2), human immunodeficiency virus–related infections (n = 2), other diagnoses (n = 7), and unknown diagnosis (n = 51). Five cases (4%) and four controls (3%) died during hospital admission.

The *rrs* LAMP result was positive in 58 of 133 patients (DSe = 43.6, 95% CI = 35.0–52.5), and 22 of 133 controls (DSp = 83.5, 95% CI = 76.0–89.3). The diagnoses for the 22 positive controls were scrub typhus (8), dengue (2), enterococcal septicemia (1), murine typhus (1), tuberculosis (1), and unknown (9). The duration of symptoms prior to presentation was significantly different between *rrs* LAMP-positive cases and *rrs* LAMP-negative cases (median = 3 days, IQR = 2–4 days, range = 1–8 days versus median 4 days, IQR = 3–6 days, range = 1–12 days, respectively; *P* < 0.001).

### Clinical evaluation of *lipL41* LAMP and comparison with *rrs* LAMP.

The *lipL41* LAMP result was positive in 50 of 133 cases, (DSe = 37.6, 95% CI = 29.3–46.4), and 13 of 133 controls (DSp = 90.2, 95% CI = 83.9–94.7). The diagnoses for these 13 positive controls were scrub typhus (4), dengue (2), eosinophilic meningitis (cause unknown) (1), murine typhus (1), and unknown (5). The differences in DSe and DSp values between the *rrs* and *lipL41* LAMP assays did not reach statistical significance (*P* = 0.13 and 0.06, respectively).

Concordance between the two assays was as follows. For cases, 43 were positive by both assays, 15 were positive by *rrs*, and 7 were positive by *lipL41*. For controls, 8 were positive by both assays, 14 were positive by *rrs*, and 5 were positive by *lipL41*.

### Sensitivity of LAMP assays in patients with leptospiremia.

Sensitivity was reanalyzed for 39 patients who were culture positive for *Leptospira* spp. The *rrs* LAMP result was positive in 33 patients (sensitivity = 84.6, 95% CI = 69.5–94.1), and the *lipL41* LAMP result was positive in 29 patients (sensitivity = 74.4, 95% CI = 57.9–87.0) (*P* = 0.29). Six culture-positive patients were negative by both assays, and all culture-positive and LAMP-negative patients were infected with *L. interrogans* serovar Autumnalis, the dominant cause of leptospirosis in Thailand during the study period.^21^

## Discussion

The ease with which LAMP can be performed to detect pathogens in clinical samples has led to its development for major infectious diseases in the developing world, including tuberculosis and malaria.^22^,^23^ Most of the published literature on LAMP describes assay development and analytical sensitivity and specificity, but relatively few studies have reported clinical evaluation of DSe and DSp. We developed an *rrs* LAMP assay for the diagnosis of leptospirosis and demonstrated 100% analytical sensitivity and specificity, and proceeded to a clinical evaluation by using samples obtained from a cohort of consecutive patients with an acute febrile illness who came to a hospital in northeastern Thailand. Having identified that the frequency of laboratory proven leptospirosis for this group was 18%, we elected to use a case–control design on the basis of cost. The DSe of *rrs* LAMP was disappointingly low (43.6%). The sensitivity of *rrs* LAMP for a subset of 39 patients who were culture positive for *Leptospira* spp. was considerably higher (84.6%). However, this observation has little practical utility because it is not possible to predict those patients with leptospiremia on the basis of clinical features alone.

A LAMP assay specific for *lipL41* has been developed previously for the diagnosis of leptospirosis and was included in our clinical evaluation. Possible reasons for the lower diagnostic sensitivity of *lipL41* LAMP compared with *rrs* LAMP include the difference in lower limit of detection for the two assays (10 GE/reaction and 100GE/reaction for *rrs* and *lipL41*, respectively), or infection with intermediate group *Leptospira* spp., which would not be detected by *lipL41*. However, the latter possibility is not the case because all 39 isolates from patients who were culture positive have been identified and none belonged to the intermediate group.

There are several possible explanations for the low DSe of both LAMP assays. A wide range of oral antimicrobial drugs are available over the counter in Thailand, and it is common for persons to self-medicate in the period leading up to hospital admission. Many of the available antimicrobial drugs would be predicted to be effective against *Leptospira* spp., which may lead to a false-negative LAMP result although not necessarily a negative MAT result. We were aware that 14 case-patients had received an antimicrobial drug at the time of admission (of which 4 case-patients had positive results for both assays), but use of a drug may have been more common. The LAMP assays may have had false-negative results because the number of organisms in the sample was below the lower limit of detection for the assay, or because of failure of primer annealing, although we consider this unlikely for *rrs*, given the conserved nature of the target. Although further refinements of the LAMP assay may result in an improvement in the lower limit of detection and diagnostic sensitivity, we are pessimistic that molecular tests will prove highly sensitive in unselected patients in settings where antibiotic use is uncontrolled.

The DSp of both LAMP assays were lower than anticipated, and it is possible that some of the LAMP-positive controls showed false-negative results by culture and MAT. The sensitivity of MAT is reported to be greater than 90%,^24^ and the finding in our study that 18 cases were culture positive but MAT negative highlights the imperfect sensitivity of MAT in our setting. The observation that 8 controls (with a diagnosis of scrub typhus [3], dengue [2], and unknown [3]) were positive by *rss* and *lipL41* assays is a further hint that these patients may have been leptospiremic. However, in the absence of definitive evidence to support true infection rather than another explanation such as laboratory contamination, we must assume that these cases had false-positive results rather than true positive results.

In conclusion, we have demonstrated that the *rss* LAMP can identify approximately half of patients with leptospirosis at the time of presentation in northeastern Thailand, but this finding is tempered by an imperfect specificity. We are unable to recommend use of LAMP in routine clinical practice until the results of additional clinical evaluations become available.

## Figures and Tables

**Table 1:** *Leptospira* spp. used in the study, Thailand*

Species	Serogroup	Serovar	Strain
*L. interrogans*	Autumnalis	Autumnalis	L0551
*L. interrogans*	Autumnalis	Autumnalis	Akiyami A
*L. interrogans*	Bataviae	Bataviae	Swart
*L. interrogans*	Djasiman	Djasiman	Djasiman
*L. interrogans*	Hebdomadis	Hebdomadis	Hebdomadis
*L. interrogans*	Icterohaemorrhagiae	Lai	Lai
*L. kirshneri*	Grippotyphosa	Grippotyphosa	Moskva
*L. kirschneri*	Cynopteri	Cynopteri	3522C
*L. borgpetersenii*	Ballum	Ballum	Mus 127
*L. borgpetersenii*	Javanica	Javanica	Veldrat Batavia 46
*L. santarosai*	Autumnalis	Alice	Alice
*L. alexanderi*	Manhao	Manhao3	L60
*L. noguchii*	Autumnalis	Fortbragg	Fort Bragg
*L. weilii*	Celledoni	Celledoni	Celledoni
*L. weilli*	Sarmin	Sarmin	Sarmin
*L. alstonii*	Ranarum	Pingchang	80-412
*L. inadai*	Lyme	Lyme	10
*L. biflexa*	Semaranga	Patoc I	Patoc I
*L. wolffii*	Khorat	Khorat	H2
*L. meyeri*	Ranarum	Ranarum	Iowa City Frog
*L. meyeri*	Semaranga	Semaranga	Veldrat Semaranga 173
*L. wolbachii*	Codice	Codice	CDC
*L. terpstrae*	Icterohaemorrhagiae	Hualin	LT11-33
*L. yanagawae*	Semaranga	Saopaulo	Sao Paulo

* Isolates were obtained from the World Health Organization/Food and Agriculture Organization of the United Nations/World Organisation for Animal Health Collaborating Centre for Reference and Research on Leptospirosis, Brisbane, Queensland Australia; the Bureau of Emerging Infection Disease, Ministry of Public Health, Bangkok, Thailand; the American Type Culture Collection, Manassas, Virigina; the Royal Tropical Institute (KIT), Amsterdam, The Netherlands; and Dr. Thareerat Kalambaheti (Mahidol University, Bangkok, Thailand).
